# Efficacy of salmon GnRHa, Ovaprim® and hCG for hormonal stimulation of spermiation in the Fowler’s toad (*Anaxyrus fowleri*)

**DOI:** 10.1093/conphys/coae056

**Published:** 2024-08-21

**Authors:** Erin M Saylor, Andrew J Kouba, Melanie R Boudreau, Nucharin Songsasen, Carrie K Kouba

**Affiliations:** Department of Biochemistry, Molecular Biology, Entomology, and Plant Pathology, 32 Creelman St., Mississippi State University, Mississippi State, MS 39762, USA; Department of Wildlife, Fisheries and Aquaculture, 775 Stone Blvd, Mississippi State University, Mississippi State, MS 39762, USA; Department of Wildlife, Fisheries and Aquaculture, 775 Stone Blvd, Mississippi State University, Mississippi State, MS 39762, USA; Smithsonian National Zoo and Conservation Biology Institute, 1500 Remount Rd, Front Royal, VA 22630, USA; Department of Biochemistry, Molecular Biology, Entomology, and Plant Pathology, 32 Creelman St., Mississippi State University, Mississippi State, MS 39762, USA

**Keywords:** amphibian, assisted breeding, domperidone, dopamine antagonist, exogenous hormones, induced spermiation, sperm aggregation

## Abstract

*Ex situ* amphibian populations can experience reproductive dysfunction due to the absence of environmental cues that trigger reproductive events. Assisted reproductive technologies (ART) for amphibians, specifically exogenous hormone regimens, can circumvent these external signals to induce gametogenesis and gamete release. Currently, the use of the mammalian reproductive hormones gonadotropin-releasing hormone (GnRH) and human chorionic gonadotropin (hCG) are used in a species-specific manner to stimulate amphibian breeding. Hormones or hormone mixtures that are effective in all breeding scenarios would provide the best option for conservation practitioners and some commercial products are already in use for breeding other ectotherms. Ovaprim®, which contains salmon GnRH analogue (sGnRHa) and the dopamine antagonist domperidone (DOM), is effective in fish aquaculture and may be effective for amphibians. To test this hypothesis, we treated Fowler’s toads (*Anaxyrus fowleri*) with either sGnRHa alone, a high or low dose of Ovaprim® or hCG. We then compared spermiation response, sperm quantity and quality parameters, and changes in animal mass over time within each treatment. We found administration of Ovaprim® resulted in more males producing sperm with better motility compared to administration of sGnRHa alone. In addition, the Ovaprim® and sGnRHa treatments resulted in lower response rates, lower sperm motilities, more abnormal sperm, and higher aggregations of sperm compared to the hCG treatment. Furthermore, Ovaprim®-treated males gained significant mass, suggesting an anti-diuretic effect of DOM. Together, these results show that neither Ovaprim® nor sGnRHa, at the concentrations tested, are likely suitable replacements for hCG in *ex situ * bufonid breeding programmes and that hormone mixtures developed for fish may have limited transferability to new world toad species.

## Teaser

This study showed that hCG is better for stimulating sperm production, quantity and quality than two hormones commonly used in fish, including salmon GnRH and salmon GnRH + domperidone (Ovaprim). These results suggest that there may be limited transferability of fish hormones to amphibians within the bufonid Bufonidae family.

## Introduction

Most temperate amphibians begin the process of reproduction in response to abiotic environmental cues such as temperature, photoperiod, barometric pressure or rainfall (Rastogi *et al.*, 2011; Smaga *et al.*, 2022), which are difficult to replicate in *ex situ* breeding programmes. Thus, captive populations of amphibians often exhibit suboptimal breeding behaviours such as calling or amplexus, poor synchronization of gamete release, low fecundity and depressed fertilization rates—all of which compromise reproductive output and the ability to maintain genetic and demographic sustainability of *ex situ* amphibian populations (Kouba *et al.*, 2009, 2012, 2013; Kouba and Vance, 2009; Clulow *et al.*, 2014; Silla and Byrne, 2019; Della Togna *et al.*, 2020). The impact of low reproductive output and potential for inbreeding can have cascading effects on reintroduction and translocation strategies, decisions on whether to supplement captive stock with new wild founders, or even whether captive breeding programmes are a viable approach for recovery of threatened species (Silla and Kouba, 2022). Additionally, collection of gametes from both captive and wild amphibian populations is necessary for biobanking and *in vitro* fertilizations (IVF) for genetic management (Kouba and Julien, 2022). Because of difficulties in breeding captive amphibians and the need to obtain gametes for genetic management, the application of assisted reproductive technologies (ART) has become commonplace for many *ex situ* amphibian populations (Bronson and Vance, 2019; Lewis *et al.*, 2019; Guy *et al.*, 2020a; Bronson *et al.*, 2021; Kouba and Julien, 2022; Burger *et al.*, 2023). Amphibian ART utilizes exogenous hormone therapies to manipulate the hypothalamic–pituitary-gonadal axis to induce breeding or obtain gametes (Bronson and Vance, 2019; Trudeau *et al.*, 2022). The most regularly used hormones in amphibian ART include the neuropeptide gonadotropin-releasing hormone (GnRH) and a mammalian gonadotropin, human chorionic gonadotropin (hCG), to stimulate sperm or egg release in both anurans and caudates (Kouba and Vance, 2009; Silla and Roberts, 2012; Marcec, 2016; Bronson and Vance, 2019; Graham and Kouba, 2022; Silla and Langhorne, 2022).

When produced endogenously, GnRH from the hypothalamus is released into the hypophyseal-portal system within the median eminence of the anuran brain and subsequently binds to receptors of gonadotropic cells in the pituitary to stimulate the synthesis and release of the gonadotropins luteinizing hormone (LH) and follicle stimulating hormone (FSH). Exogenous hormone therapy regimens using GnRH analogues (mainly GnRH; [des -Gly10, D-Ala6]-LH-RH) and the mammalian gonadotropin hCG have been used to induce spermiation in male anurans with varying rates of success by species (Browne *et al.*, 2006a, 2006b; Mann *et al.*, 2010; Trudeau *et al.*, 2010; Kouba *et al.*, 2012; Silla and Roberts, 2012; Langhorne *et al.*, 2021; Burger *et al.*, 2021; Silla and Byrne, 2021; Naranjo *et al.*, 2022). The spermiation response to these two exogenous hormones may be family- or genera-specific. For instance, Silla and Roberts (2012) showed that five Australian anuran species belonging to the family *Myobatrachidae* produced more sperm in response to GnRH administration, whereas three species belonging to the family *Lymnodynastidae* produced more sperm in response to hCG administration. Furthermore, the American toad (*Anaxyrus americanus*) produced a low concentration of sperm in response to GnRH compared to hCG (Kouba *et al.*, 2012), yet the Panamanian golden frog (*Atelopus zeteki*) produced more sperm in response to GnRH compared to hCG (Della Togna *et al.*, 2017).

In addition to optimizing gamete production, hormone therapies aim to elicit appropriate breeding behaviours, which is important for natural breeding in captivity. For most male bufonid species of the genus *Anaxyrus*, hormone treatment using GnRH more effectively elicits natural breeding behaviours, such as calling or amplexus, compared to treatments using hCG; however, hCG treatments typically result in more animals producing higher concentrations of sperm (Kouba *et al.*, 2009, 2012). As a result, to maximize the effects of both gamete production and breeding behaviours, the two hormones are often administered together (Calatayud *et al.*, 2015; Graham, 2015; Guy *et al.*, 2020a; Trudeau *et al.*, 2022). Incorporation of dopamine antagonists with GnRH treatment has been trialled in several amphibian species as dopamine has been shown to have an inhibitory effect on gonadotropin release in amphibians (Trudeau *et al.*, 2013; Vu and Trudeau, 2016; Della Togna *et al.*, 2017; Bronson *et al.*, 2021). By preventing the inhibitory action of dopamine on gonadotropin secretion the target activity on gonads may be enhanced to elicit stronger responses to hypothalamic exogenous hormone therapies (Sotowska-Brochocka and Licht, 1992; Sotowska-Brochocka *et al.*, 1994). Although blocking the action of dopamine may enhance response to hormone therapy, dopamine plays other roles in the body such as regulation of urinary flow and water trafficking in cells (Zelenina *et al.*, 2002; Pravikova and Ivanova, 2022). Thus, physiological responses to dopamine antagonists such as osmoregulatory changes are an important consideration when developing hormone regimens.

For the genus *Anaxyrus,* hCG has been established as the standard hormone used for inducing spermiation as it results in greater sperm output compared to GnRH when performing IVF procedures or when sperm is needed for biobanking genetic lineages. If the goal is to induce natural breeding, hCG is combined with GnRH because it stimulates normal reproductive behaviours and promotes spermiation leading to successful breeding events. When utilizing both hCG and GnRH in hormone therapies multiple hormones must be purchased, reconstituted, administered separately. Moreover, each hormone has strict storage requirements which can be costly and difficult for institutions to implement in breeding programmes. One potential alternative is to use commercially pre-prepared products such as Ovaprim®, which is used frequently in the aquaculture and fisheries industries for breeding brood stock.

Ovaprim®, containing salmon GnRHa (Glp-His-Trp-Ser-Tyr-DArg-Trp-Leu-Pro-NHet) plus the dopamine antagonist domperidone (DOM), was developed to enhance reproduction in captive fish brood stock. Like amphibians (Vu and Trudeau, 2016), teleost fish experience dopaminergic inhibition of reproduction (Barsagade, 2020); thus, addition of DOM to sGnRHa blocks the inhibitory action of dopamine and theoretically bolsters the response to sGnRHa. For example, male longspine scraper (*Capoeta trutta*) fish had an increased gonadosomatic index, higher volumes of released sperm and longer motility duration of sperm in response to Ovaprim® compared to treatments using hCG (Zadmajid, 2016). While the use of Ovaprim® in fish has been widely studied (Acharjee *et al.*, 2017; Pawar *et al.*, 2019; Kucharczyk *et al.*, 2020; Yeganeh *et al.*, 2022; Nargesi *et al.*, 2023) its potential applicability to amphibian breeding programmes is relatively unexplored.

Despite GnRH treatment in some amphibian species resulting in poor sperm production, the GnRH analogue [des -Gly10, D-Ala6]-LH-RH has been the most widely used GnRH for sperm and egg release in anurans yet other forms of GnRH have not been thoroughly investigated. Multiple GnRH molecules and several GnRH receptor types have been found in the anuran brain (Licht and Papkoff, 1974; Muñoz-Cueto *et al.*, 2020) at varying levels depending on the species, likely performing differing roles in regulation of reproduction. Additionally, the most abundant GnRH form found in a species is not necessarily the most potent form to endogenous receptors (Licht and Papkoff, 1974; McCreery *et al.*, 1982; Licht *et al.*, 1987, 1994; Millar, 2003, 2005; Muñoz-Cueto *et al.*, 2020). Relatively low response rates and low sperm concentration in some anuran species in response to GnRH treatment may be the result of low binding affinity of GnRH to receptor types found in these species. As sGnRHa-like molecules have been found in anuran brains (Sherwood *et al.*, 1986; King and Millar, 1995; Ebersole and Boyd, 2000), Ovaprim® or sGnRHa alone may provide more effective alternatives to GnRH when attempting hypothalamic stimulation of reproduction in amphibians. Ovaprim® may also provide a simpler, cost-efficient hormone combination that does not require reconstitution and storage of hCG and GnRH, simplifying personnel training for implementation within amphibian conservation programmes. Early application of Ovaprim® successfully induced spawning and fertilization in the tiger frog, *Hoplobatrachus occipitalis* (Godome *et al.*, 2021) but failed to induce a response in the Australian southern bell frog (*Litoria raniformis*) (Mann *et al.*, 2010)*.* In neither case was the effect of sGnRHa or Ovaprim® on sperm quality or quantity assessed. Moreover, these studies suggest that the spermiation response to Ovaprim® may differ between species, as observed for other hormone treatments in anurans.

Considering the similarities in neuroendocrine control of reproduction between teleost fish and amphibians, and the differential response of anurans to Ovaprim® in previous studies, we investigated if sGnRHa and Ovaprim® administered to Fowler’s toads (*Anaxyrus fowleri*) would stimulate spermiation and produce sperm samples of higher quality and quantity compared to male toads treated with hCG. Additionally, we explored change in mass of treated animals in response to Ovaprim® and sGnRHa to investigate potential osmoregulatory changes due to the action of DOM in Ovaprim®. To assess the efficacy of these hormones for stimulating spermiation, Fowler’s toads were administered one of five treatments: (1) Ovaprim® high dose; (2) Ovaprim® low dose; (3) sGnRHa; (4) hCG; and (5) phosphate-buffered saline (PBS) control. We measured spermiation response rate, sperm quality and quantity parameters and recorded toad mass at eight time points over a 24-h period after hormone administration.

## Materials and Methods

### Collection and care of animals

Fowler’s toads (*n* = 30) were collected in 2021 from Oktibbeha County, Mississippi, and housed at Mississippi State University Conservation Physiology Lab Mississippi State University, MS USA (Permit #; 0504202; 0415221) in accordance with IACUC protocols (IACUC #19–345; #22–335). Animals were acclimated to captive conditions for 4–6 months prior to start of experimental trials. Toads were housed in plastic polycarbonate tanks (30 cm H × 46 cm W × 66 cm L) furnished with sheet moss (Moss Unlimited, Washington USA), refugia and a water tub. Animals were fed a rotating diet of gut-loaded crickets, mealworms and dubia roaches dusted with vitamins (Rep-Cal® Herptivite and Calcium with vitamin D_3_) three times per week. Tanks were kept under UVB lamps on a 12 h light cycle and the temperature of enclosures was ~21°C throughout the year. Animals were identified as male based on the presence of dark vocal sacs, nuptial pads and sperm production and individually identified based on their unique dorsal spot patterns.

### Hormone preparation and treatment groups

All trials were performed outside of the peak breeding season (April–June) to avoid any confounding factors. Ovaprim® was obtained from Syndel company (catalogue NDC# 50378–012-01, Washington, USA) and stored at 4°C until use. Commercial Ovaprim® is marketed for intraperitoneal or intramuscular injection as a spawning aide for finfish brood stock and comes in a stock at 20 μg/ml sGnRHa +10 mg/ml DOM in a 10-ml bottle. Lyophilized sGnRHa (Glp-His-Trp-Ser-Tyr-DArg-Trp-Leu-Pro-NHet) was purchased from Creative Peptides Inc. (catalogue # OPO-011, New York, USA) and was reconstituted in PBS, aliquoted and stored at −20°C until used. Lyophilized hCG was obtained from Sigma-Aldrich (catalogue #CG5, Missouri, USA) and stored in the powder form at 4°C until needed, then reconstituted with PBS prior to administration. Males (*n* = 15/treatment) were randomly assigned to one of five treatment groups: (1) Ovaprim® high dose consisting of 6 μg sGnRHa + 3 mg DOM (= 300 μl Ovaprim®); (2) Ovaprim® low dose consisting of 1.2 μg sGnRHa + 0.6 mg DOM (= 60 μl Ovaprim®); (3) sGnRHa alone (6 μg sGnRHa), which is equivalent to the concentration of sGnRHa in the high-Ovaprim® treatment; (4) 300 IU hCG; and (5) a PBS control (300 μl) (as detailed in Table 1). In our design, we used a crossover experimental set-up, where a single animal can provide several experiment units. In this design, each animal is used as its own control and was randomly assigned two or more distinct hormone treatments, separated by washout periods of 2 months before another trial. As toads can be exposed to different treatments in different test periods, the experimental unit is the animal for a set period of time; this design helps to address the three Rs of experimental treatments on animals: Reduce, Refine and Reuse.

**Table 1:** Treatment concentrations, initial average mass, number of animals producing sperm and proportion of spermic urine samples for all urine collected over time.

**Treatment**	**Hormone concentration or international unit**	**Initial mass (g)**	**Animals producing sperm**	**Proportion of spermic samples collected over time**
**hCG**	300 IU	24.0 ± 1.23	15/15 (100%)	92/105 (88%)
**Ovaprim® high**	6 μg sGnRHa +3 mg DOM	24.0 ± 0.80	11/15 (73%)	37/83 (45%)
**Ovaprim® low**	1.2 μg sGnRHa +0.6 mg DOM	24.0 ± 0.85	7/15 (47%)	12/102 (12%)
**sGnRHa**	6 μg sGnRHa	23.7 ± 0.84	8/15 (53%)	25/104 (24%)
**Control PBS**	300 μl PBS	25.3 ± 1.01	0/15 (0%)	0/105 (0%)

Toad mass was measured prior to hormone injection. All hormones were administered intraperitoneally using syringes with sterile 30-gauge needles. Following hormone administration, toads were placed into 19.0 × 29.5 × 15.5 cm plastic holding tubs with 1 cm of aged tap water to encourage hydration through their ventral drink patch.

### Sperm collection and analysis

Urine collections occurred at 0 (prior to hormone administration), 1, 2, 3, 5, 7, 9 and 24 h post-hormone administration. To collect urine, males were quickly removed from their container, patted dry with Kimwipes® and held cloaca facing down into a sterile petri dish with their legs held apart. Toads typically urinated into dishes within 1 min of restraint, as part of their natural defence mechanism. Urine was immediately assessed for sperm presence, motility and the proportion of morphologically normal sperm determined under a phase contrast microscope (Olympus CX43; 40× magnification), and the remaining spermic urine was stored at 4°C. All toads were aspermic prior to hormone treatment (Hour 0). After each urine collection, toads were weighed to monitor any changes in water retention over time.

The presence of sperm in a urine sample indicated a spermiation response. The latency to spermiation and duration of spermiation varied considerably across individuals and across treatment groups, with some males releasing sperm at multiple hours but others only released sperm at a single hour. The spermiation response was analysed in two ways: 1) by assessing the proportion of total individuals with a spermic response at any collection point (herein called ‘spermiation across individuals’); and 2) the total number of spermic urine samples to total urine samples (herein called ‘spermiation across samples’) collected post-hormone administration (after Hour 0), within each treatment. If an individual did not provide a urine sample, the individual was excluded from the analysis for that hour. Peak response was determined by assessing the time points at which the greatest number of males responded within each treatment. Sperm motility was measured immediately after collection. Sperm were categorized as forward motile (FM) if flagellar movement was observed and the sperm progressed forward, stationary motile (SM) if there was flagellar movement but no forward progression or non-motile (NM) if no flagellar movement was observed. Sperm morphology was measured by counting the number of sperm exhibiting an abnormal head or tail (broken, bent, missing or double heads or tails). To measure sperm concentration, 10 μl of each sperm sample was diluted with hyperosmotic PBS (275–304 mOs/kg) for arresting sperm motility and counted on a Neubauer hemacytometer. Due to frequently observed aggregation of sperm in some treatments, select samples (*n* = 2) were stained with SYBR and propidium iodide (Burger *et al.*, 2022) to determine the presence of live sperm in the aggregated sperm.

**Figure 1:**
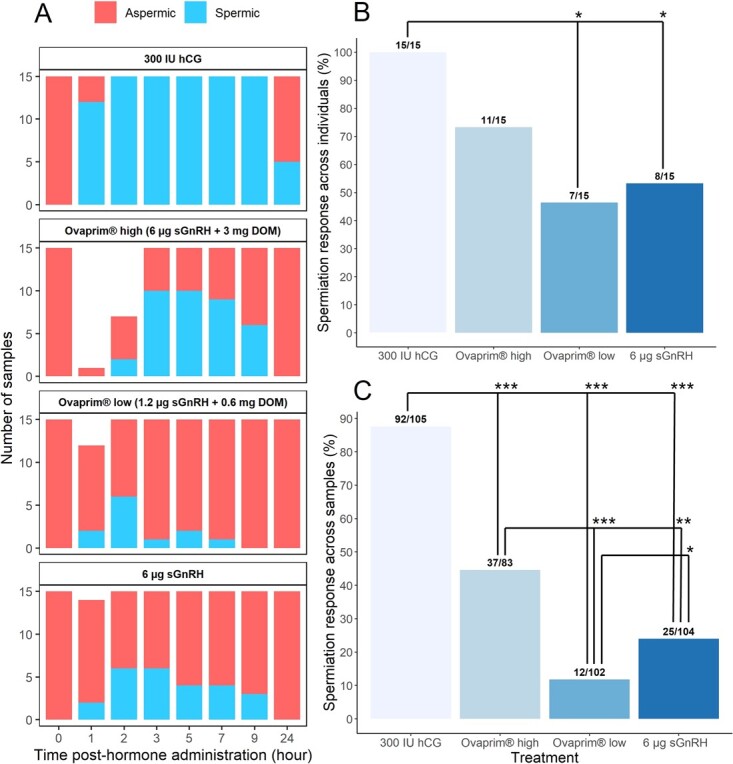
Panel A shows the proportion of urine samples containing spermatozoa over time for treatments: 1) 300 IU hCG; 2) 300 μl Ovaprim®; 3) 60 μl Ovaprim®; and 4) 6 μg sGnRHa. Panel B shows the spermiation response rate for the total number of individuals in each treatment, whereas Panel C shows the sperm production response rate for the total number of samples collected for each treatment ( ^*^ *P* < 0.05, ^**^ *P* < 0.01, ^***^ *P* < 0.001).

### Statistical analysis

Fischer’s exact tests were used to assess overall spermiation response across individuals and samples between treatments; *P-*values with Odds Ratios (OR) are reported. Due to lack of samples collected at certain time points for some treatments, and non-normal distributions of data, a Generalized Additive Mixed Effects Model with Location Space and Scale (GAMLSS) was used to assess effects between treatments and time on both sperm motility and abnormality. A zero-inflated beta (BEZI) distribution was used with individual animal run as a random factor to eliminate the effect of individual variation in response. The changes in mass were assessed for normality by visual inspection of residual plots and a Shapiro–Wilk test and checked for heteroskedasticity using Levene’s test. Due to heterogeneity of variances and slight non-normality, a weighted least squares regression model was used to analyse the effect of treatment over time on changes in mass, with individual as a random factor. Data are presented as Means ± SEM, significance was set at *P* < 0.05 and statistical analyses were performed using R statistical programming software v. R 4.2.3 (R Core Team, 2023).

## Results

### Spermiation response across treatments

All males provided a urine sample at every hour in the hCG (Fig. 1A) and control treatments. In the Ovaprim®-high treatment, urine could not be collected from 14 and 8 males at the 1 and 2 h time point, respectively (Fig. 1A). In the Ovaprim®-low group, 3 males could not be collected at the 1-h time point (Fig. 1A). In the sGnRHa treatment, 1 male did not provide a sample at the 1 h time point (Fig. 1A). The greatest number of males producing sperm occurred at the: 1) 3 and 5 h time points (67%; *n* = 10/15) for the Ovaprim® high treatment; 2) 2 h time point (40%; *n* = 6/15) for the Ovaprim® low treatment; and 3) 2 and 3 h time points (40%; 6/15) for the sGnRHa treatment (Fig. 1A). In contrast, 80% (*n* = 12/15) of the hCG treated males were producing sperm at the 1 h time point and 100% of the animals were producing sperm between the 2 and 9 h time points (Table 1).

Spermiation response across individuals (i.e. total number of males producing sperm throughout trials to total number of males in treatment) with Fischer’s exact tests revealed no significant difference between hCG and the Ovaprim® high treatment (Fig. 1B). Spermiation across individuals for the Ovaprim® low and sGnRHa treatments were significantly lower compared to the hCG treatment (OR < 0.001, *P <* 0.001 and 0.001, respectively; Fig. 1B), but not significantly different compared to the Ovaprim® high treatment (Fig. 1B). When spermiation response was analysed as spermiation across samples (i.e. total number of spermic samples to total samples per treatment) with Fischer’s exact tests, spermiation was significantly lower in Ovaprim® and sGnRHa treatments (OR = 0.02–0.11, *P* < 0.05 across treatment comparisons; Fig. 1C) compared to the hCG group. In addition, spermiation across samples was significantly greater in the Ovaprim® high treatment compared to both the sGnRHa (OR = 2.40, *P* < 0.001) and the Low Ovaprim® treatments (OR = 5.19, *P* < 0.001), with the sGnRHa significantly greater (OR = 0.46, *P* = 0.04) than the Ovaprim® low treatment.

### Sperm aggregation

**Figure 2:**
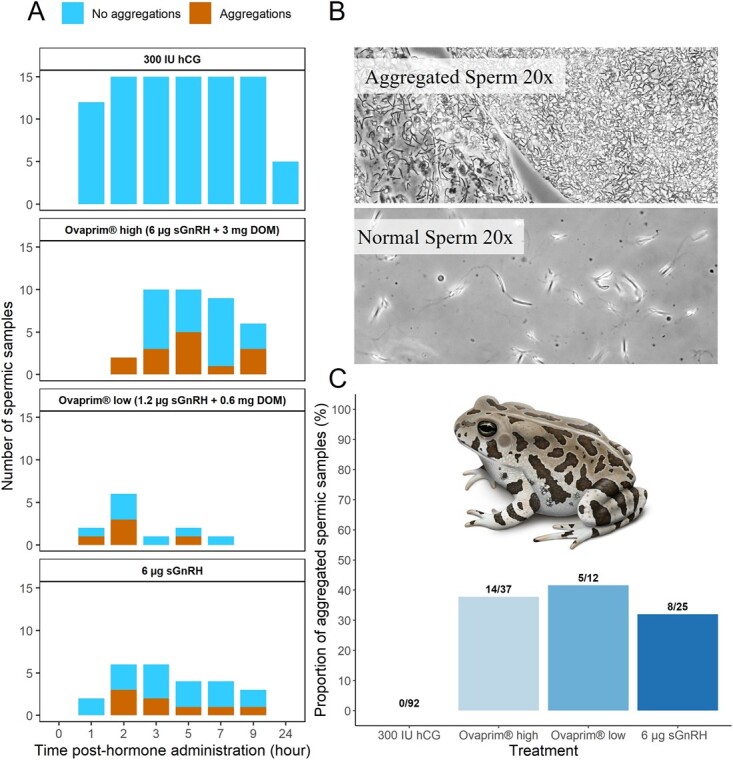
Panel A shows the proportion of spermic samples that contained aggregated sperm over time for each treatment: 1) 300 IU hCG; 2) 300 μl Ovaprim®; 3) 60 μl Ovaprim®; and 4) 6 μg sGnRHa. Panel B shows a comparison of sperm aggregated in the sample from the Ovaprim® high treatment group to normal sperm in a sample from a male treated with hCG. Panel C shows the frequency of samples exhibiting sperm aggregation for each treatment group.

Sperm aggregation occurred in both Ovaprim® treatments and the sGnRHa group (Fig. 2). The highest frequency of samples exhibiting sperm aggregation occurred in the Ovaprim® low treatment (41.6%), followed by the Ovaprim® high (37.8%) and the sGnRHa cohorts (32.0%). All sperm that were aggregated appeared non-motile. However, live/dead staining with propidium iodide and SYBR14 revealed many sperm were alive despite aggregation (data not shown). Analysis by Fisher’s exact test showed no difference (OR = 0.8–1.2, *P* > 0.7) in frequency of samples exhibiting sperm aggregation amongst the Ovaprim® high, Ovaprim®low and sGnRHa treatments (Fig. 2). It is also notable that aggregated sperm was not observed in any samples collected from the hCG treatment (Fig. 2).

### Sperm Motility, Concentration, and Morphology

**Figure 3:**
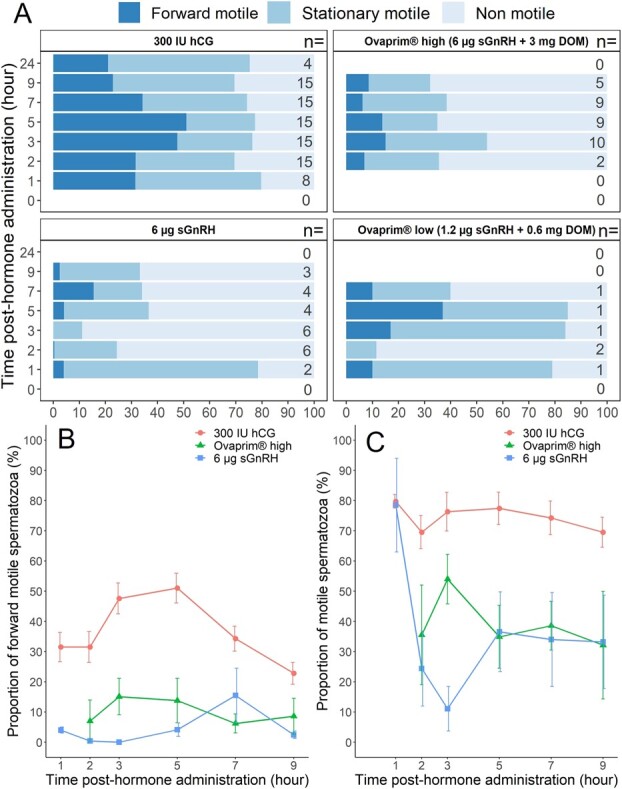
Panel A shows the proportions of forward motile sperm (FM), stationary motile sperm (SM) and non-motile sperm (NM) in urine samples over time (0–24 h) within each treatment, with the number of samples analysed listed on the right. Panel B shows the average proportion of sperm with forward motility (FM) over time between treatments, whereas Panel C shows the average proportion of sperm exhibiting motility (FM + SM) in samples over time between treatments. All data are presented as Means ± SEM.

**Figure 4:**
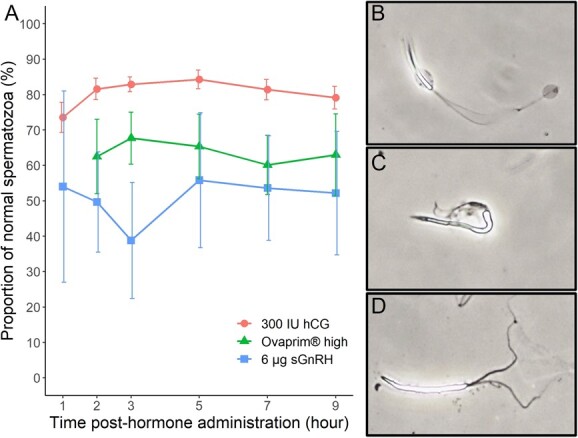
Panel A shows the average proportion of morphologically normal spermatozoa produced over time (0–24 h) across treatments. Data are presented as Means ± SEM. Panel B shows a morphologically normal sperm under 20× magnification. Panel C and D show an abnormal spermatozoa with misshapen head and tail or split tail, respectively, under 20× magnification.

Sperm aggregation in both Ovaprim® treatments and the sGnRHa group (Fig. 2) prevented accurate calculation of sperm concentration. Due to the low number of males producing sperm in the Ovaprim® low treatment (Fig. 2A), this treatment was excluded from the GAMLSS analysis of sperm motility and morphology. There was no difference in forward or total motility between the hCG and the Ovaprim® high treatments. However, compared to the hCG treatment, sGnRHa resulted in significantly lower total motility (F_29,113_ = 0.39, *P* < 0.001) and forward motility (F_23,119_ = 3.67, *P* < 0.001) across time (Fig. 3). Total motility in the sGnRHa group was also significantly lower (F_30,113_ = 20.16, *P <* 0.001) compared to the Ovaprim® high group over time, and there was also a marginal difference (F_23,119_ = 20.16, *P* = 0.08) in forward motility between these two treatments, with forward motility being slightly higher in the Ovaprim® high group (Fig. 3B and C). We also observed a significant effect (F_29,113_ = 6.04, *P* = 0.01) of time on total motility in the Ovaprim® high treatment, with variation in motility across time points (Fig. 3B and 3C). There were fewer morphologically normal sperm in the Ovaprim® high and sGnRHa treatments compared to the hCG treatment (F_29,106_ = 5.1 and 12.32, respectively, *P* < 0.001 both cases; Fig. 4) over time. Although the Ovaprim® high treatment resulted in more normal sperm on average throughout time compared to sGnRHa alone (Fig. 4), this difference was not significant and there were no significant effects of time on sperm morphology in any treatment, nor was there an interaction between treatment and time.

### Change in Mass

**Figure 5:**
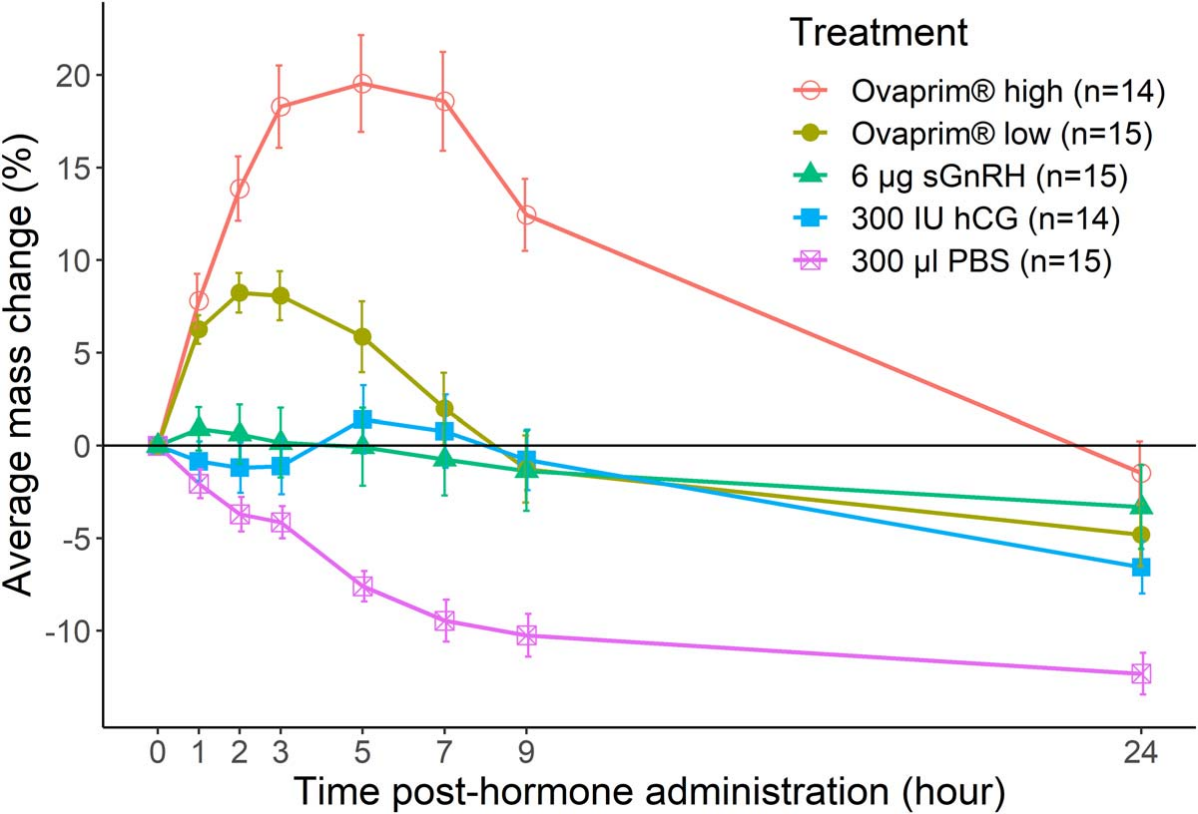
Average proportional mass change of individuals over time (0–24 h) across the five treatments: 1) Ovaprim® high; Ovaprim® low; 3) sGnRHa; 4) hCG; and 5) control. Data are presented as Means ± SEM.

There was very little change in the mass of males treated with hCG, which remained close to 0, or baseline, throughout the collection period (Fig. 5) and is comparable to previous data (McDonough *et al.*, 2016). However, males treated with the high dose of Ovaprim® gained 19.5% mass on average, peaking at Hour 5 and then returning to normal (0%) at 24 h post-hormone administration (Fig. 5). The change in mass for the Ovaprim® high treatment was significantly greater (F_39,544_ = 11.94–41.86, *P* < 0.001 across time) than change in mass for the hCG treatment across all hours, except Hour 24. Increases in mass were also observed in the Ovaprim® low treated toads (+8.2%), which peaked earlier at Hour 2 and diminished to −4.8% of their initial mass after 24 h (Fig. 5). The Ovaprim® low treatment also caused significantly greater (F_39,544_ = 0.02–9.39, *P* < 0.05 across time points) changes in mass compared to the hCG treatment at Hour 1, 2 and 3 (Fig. 5). Treatment with sGnRHa did not result in a significant change in mass at any time point compared to the hCG treatment (Fig. 5). Control males treated with PBS showed significant (F_39,544_ = 4.63–13.06, *P* < 0.05) loss of mass at Hour 5, 7, 9 and 24 compared to males treated with hCG (Fig. 5).

## Discussion

### Effects of Ovaprim® on spermiation response and sperm quality

Understanding the effect of different hormones and dopamine antagonists for induction of spermiation and optimizing quantity and quality of sperm produced is important for the successful and efficient propagation of amphibian brood stock when using ART. Both Ovaprim® and its component sGnRHa induced spermiation yet resulted in lower spermiation rates and produced sperm with greater abnormalities and lower motility compared to hCG in the bufonid species *A. fowleri*. Additionally, due to many toads not providing sperm at all collection hours and latency to spermiation differing between individuals within treatments, peak spermiation response in non-hCG treatments differed widely between groups and between individuals within the same cohort.

The effects of hCG on spermiation seen in this study align with effects previously documented for certain taxa including *Bufonidae* and *lymnodynastidae*, where hCG treatments are generally more effective compared to GnRHa ([des-Gly10, D-Ala6]-LH-RH) (Kouba *et al.*, 2012; Silla and Roberts, 2012; Uteshev *et al.*, 2012). Other studies investigating hormonal induction of spermiation in *Anaxyrus sp.* (e.g. American toads; *A. americanus*) show that animals administered GnRHa have lower rates of spermiation and lower sperm concentration, but similar sperm motility, compared to hCG (Kouba *et al.*, 2012). While tThe Ovaprim® high and sGnRHa treatments in this study elicited lower response rates and poorer sperm quality compared to hCG, these treatments resulted in greater spermiation response rates in *A. fowleri* compared to treatments of GnRHa documented in *A. americanus* (73.3% and 53.3%, respectively, vs. 35% with GnRHa; Kouba *et al.*, 2012). The lower number of males responding to GnRHa in another *Anaxyrus* species suggests that the sGnRHa isoform may produce a greater response rate in this genus.

The differential response to GnRH isoforms and analogues may be due to the potency of the molecules varying by genera and between receptor types due to ligand selectivity. For example, in the bullfrog (*Lithobates catesbeianus*), chicken GnRH has a higher potency for all three GnRH receptors found in the bullfrog brain compared to sGnRHa and mammalian GnRH (Wang *et al.*, 2001). Many species of anurans that do not respond well to either GnRHa or hCG, such as the southern bell frog (*Litoria raniformis*; (Mann *et al.*, 2010), may respond better to other forms of GnRH, including sGnRHa or chicken GnRH. In addition to poor receptor–ligand specificity of some hormones, sub-optimal routes of hormone administration may play a role in poor response to hormone treatment. For example, intraperitoneal administration of GnRH elicits low spermiation rates compared to hCG in bufonids , but intranasal administration of GnRH to male *A. fowleri* elicits spermiation in up to 95% of males—likely due to the proximity of hormone administration to receptors in the brain (Julien *et al.*, 2019). Assessment of spermiation response to intranasal administration of sGnRHa would provide more insight into the applicability of sGnRHa for hormonal induction of spermiation in anurans. In both *A. americanus* and *A. fowleri*, intraperitoneal administration of hCG elicits a greater response rate and higher quality sperm compared to intraperitoneal administration of sGnRHa (shown here), GnRHa (Kouba *et al.*, 2012) and intranasal administration of GnRHa (Julien *et al.*, 2019).

In addition to the lower spermiation response, sperm motility and poorer morphology found in our Ovaprim® and sGnRHa treatments, sperm aggregation was also frequently observed in these groups. In anurans, sperm develop in spermatogenic cysts, which travel through the testes lobule and are released by Sertoli cells through pathways dependent on NA+/K+ ion pumps (Volonteri and Ceballos, 2010). Aggregations were seen in both the Ovaprim® and sGnRHa groups but not in the hCG treatment, and aggregations have not been reported for GnRHa. In fish and mammals, GnRH peptides binding to receptors in the gonads have been implicated in paracrine signalling pathways in regulation of gonadal function (Gazourian *et al.*, 1997; Ramakrishnappa *et al.*, 2005), with sGnRHa causing germinal vesicle breakdown in zebrafish ovaries (Fallah and Habibi, 2020). In the amphibian, *Rana esculenta,* GnRH applied to testes *in vitro* enhanced primary spermatogonia multiplication (Minucci *et al.*, 1986). Here, administration of exogenous sGnRHa binding to receptors on the gonads may cause a dysfunction in the release of sperm from spermatogenic cysts; however, more research is needed to confirm the exact cause of sperm aggregation.

Despite being viable, aggregated sperm produced through Ovaprim® or sGnRHa may not have optimal fertilizing capacity, as motility of sperm has been shown to be important for fertilization success (Dziminski *et al.*, 2009). Additionally, if sperm is being collected for the purpose of cryopreservation, genetic management and IVF procedures, samples must contain an adequate concentration of spermatozoa. Because concentration of aggregated samples cannot be measured, sperm aggregations present in samples impede the use of spermic urine for both cryopreservation and IVF. Considering the lower response rate, lower sperm motilities, higher sperm abnormalities and aggregation of sperm obtained from Fowler’s toads treated with Ovaprim® compared to sperm obtained using hCG, Ovaprim® does not provide a better alternative to hCG for exogenous hormone treatment of *A. fowleri* to elicit spermiation.

### Influence of DOM

Significantly greater spermiation response, higher sperm motility and greater % of normal sperm were obtained in the Ovaprim® high treatment compared to treatment with sGnRHa alone. These findings support existing evidence that dopamine antagonists combined with hypothalamic hormones elicits a more effective spermiation response and the release of higher quality sperm compared to treatments of hypothalamic hormones alone in anurans (Trudeau *et al.*, 2010; Vu *et al.*, 2017). Consistent with our results, combinations of GnRH with either DOM or a different dopamine antagonist (i.e. metoclopramide) were both effective for inducing spawning in *Lithobates pipiens* (Trudeau *et al.*, 2013).

Considering Ovaprim® is designed for use in fish aquaculture, the ratio of the sGnRHa and dopamine antagonist in the mixture is specifically formulated for exogenous hormone treatment in fish. Manipulation of the ratio of sGnRHa to dopamine antagonist for amphibian hormone treatment to stimulate a hormone cascade more specific to anuran endocrine systems may increase the efficacy of spermiation treatments using sGnRHa, particularly in species that may respond well to the salmon GnRH isoform. Results from this study show that Ovaprim®, as formulated, has low transferability from fish to amphibians. In particular, the 53% response rate to sGnRHa alone warrants further investigation at higher concentrations for *Anaxyrus* and application to other genera that do not respond well to GnRHa. Furthermore, adjusting the concentration of dopamine antagonists when combined with sGnRHa may reduce the osmoregulatory changes observed here.

### Physiological effects of hormone treatment

Difficulties in obtaining urine samples from Ovaprim® treated males within the first 2 h post-administration prevented the acquisition of spermic samples and presents a disadvantage of using Ovaprim® in *A. fowleri.* This observation may be related to the rapid increase in mass of the treated individuals. Additionally, mortality was seen in two males in the Ovaprim® high treatment group, and necropsy of deceased animals revealed large amounts of fluid in the coelomic cavity, indicating a strong anti-diuretic effect of Ovaprim®. Because mass gain was not seen in animals treated with sGnRHa alone, DOM may be responsible for the fluid retention. Osmoregulation in anurans is controlled by complex interactions of cellular signalling and water channel proteins embedded in cellular membranes called aquaporins (Suzuki *et al.*, 2015). Fowler’s toads possess a drink patch located on the pelvic portion of their ventral area—an area wherein several types of aquaporins have been found in other anuran species. For example, in *Xenopus laevis*, an aquaporin 3 homologue exists within granular and mucous glands of skin (Mochida *et al.*, 2008), and a homologue to mammalian aquaporin 2 is found in the ventral skin of *Hyla japonica* (Hasegawa *et al.*, 2003). There are also specific aquaporins specialized for osmoregulation within the kidneys, yet other types are specialized for osmoregulation via water movement through the skin, such as aquaporin -h2 and aquaporin -h3 (Mochida *et al.*, 2008). Thus, changes in osmoregulation seen in Fowler’s toads may occur through aquaporins in the skin or in other areas of the body such as the kidneys. Aquaporin activation pathways in anuran cells have been shown to be regulated by hormones such as arginine vasotocin, which acts as an anti-diuretic in the kidneys (Bentley, 1969) and increases water permeability in anuran skin (Saitoh *et al.*, 2014). In previous research, use of arginine vasotocin for exogenous hormone treatment resulted in a similar retention of fluid and some mortality in *A. fowleri* (Julien, 2021). While the influence of dopamine on osmoregulation via aquaporins has not been researched in amphibians, in mammalian renal cells, dopamine and protein kinase C can decrease the water permeability of aquaporin 4 (Zelenina *et al.*, 2002). Considering the possibility that dopamine may influence aquaporin activation pathways, administration of dopamine antagonists may result in changes in osmoregulation of treated animals through aquaporin regulation in the drink patch or within the kidneys. The anti-diuretic effect of the Ovaprim® high treatment further validates the recommendation that if DOM and sGnRHa were to be used together, the ratios would need to be adjusted to maintain the higher sGnRHa concentration, whilst lowering the DOM component.

### Economic cost comparison of hormone treatments

To evaluate the economic viability of the Ovaprim® and sGnRHa treatments tested in this study as options for hormone therapy in anuran breeding compared to GnRHa and hCG, the cost of hormones purchased for this study are outlined in Table 2. Salmon GnRHa has the highest cost per unit, followed by hCG, Ovaprim® and GnRH. The cost was further assessed by dividing the per unit cost by the approximate number of *A. fowleri* that can be treated per unit to obtain a cost per animal for each hormone. hCG has the highest cost per animal ($10.30) because of the large amount of hCG that is required to stimulate spermiation, while GnRH has the lowest cost per animal ($0.90). The cost per animal for Ovaprim® ($1.57) is based on the Ovaprim®-high treatment dosage used in this study because this group had significantly more animals responding compared to the lower dose of Ovaprim®. Cost per animal is similar between Ovaprim® and sGnRHa alone, with sGnRHa being slightly lower ($1.20). Not included in this cost breakdown are the costs of reagents, laboratory equipment and personnel training required for reconstitution and storage of lyophilized hCG, GnRH and sGnRHa, which could present limitations for application in captive breeding efforts, but are otherwise similar across all these treatments.

**Table 2:** Comparative cost of spermiation dosages of hCG, GnRH, Ovaprim® and sGnRHa for Fowler’s toads weighing ~24 g.

	hCG	GnRH	Ovaprim®	sGnRHa
Company	Sigma	Sigma	Syndel	Creative Peptides
Catalogue #	CG5	L4513	OVAM-M-ML-U010FN	OPO-011
Per bottle price (USD)	$165.00	$91.00	$52.00	$199.60
Hormone quantity per bottle	5000 IU	1000 μg	200 μg sGnRHa +100 mg DOM	1000 μg
Dosage (for male *A. fowleri* spermiation)	300 IU	0.4 μg/g BW	6 μg sGnRHa +3 mg DOM	6 μg
# *A. fowleri* treated per unit (for ~24-g male)	16	104	33	166
Cost/animal (USD)	$10.30	$0.90	$1.57	$1.20

## Conclusions

The Amphibian Conservation Action Plan lists *ex situ* conservation initiatives such as captive breeding and biobanking as imperatives to address the amphibian extinction crisis (IUCN SSC Amphibian Specialist Group, 2005; 2022). Amphibian *ex situ* conservation relies on successful breeding, genetic management and transfer of genetic material between captive and wild populations, which are often difficult or impossible to achieve without the development and optimization of exogenous hormone therapies. Development of hormone regimens that successfully elicit responses in both male and female amphibians across species is an important research objective to provide a cost-effective, easy approach to amphibian assisted breeding or as a method of obtaining gametes for biobanking genetic lineages. This study is the first to find that exogenous sGnRHa alone elicits spermiation in anurans and one of the first to assess Ovaprim® as a hormone for amphibian ART in a bufonid. Although Ovaprim® or sGnRHa alone are less costly than hCG, the low sperm motility, abnormal sperm, anti-diuretic effect of DOM and aggregation of sperm present trade-offs to the lower cost per animal of Ovaprim® compared to hCG at the concentrations tested here. While sGnRHa and Ovaprim® are less costly than hCG, the latter is still the more effective hormone for sperm production, quantity and quality in bufonid species.

## Data Availability

Data is available upon request.
